# Analysis of β-Galactosidase During Fruit Development and Ripening in Two Different Texture Types of Apple Cultivars

**DOI:** 10.3389/fpls.2018.00539

**Published:** 2018-04-24

**Authors:** Huijuan Yang, Junling Liu, Meile Dang, Bo Zhang, Hongguang Li, Rui Meng, Dong Qu, Yazhou Yang, Zhengyang Zhao

**Affiliations:** ^1^State Key Laboratory of Crop Stress Biology in Arid Areas, College of Horticulture, Northwest A&F University, Yangling, China; ^2^Shaanxi Province Key Laboratory of Bio-Resources, School of Biological Science and Engineering, Shaanxi University of Technology, Hanzhong, China; ^3^Apple Engineering and Technology Research Center of Shaanxi Province, Northwest A&F University, Yangling, China

**Keywords:** apple, β-galactosidase, texture, fruit ripening, firmness

## Abstract

β-galactosidase (β-Gal), one of the cell wall modifying enzymes, plays an important role in fruit ripening and softening. However, its role in apple fruit texture remains unclear. In this study, the role of β-Gal was analyzed in two apple cultivars, ‘Fuji’ and ‘Qinguan,’ which are characterized by different fruit texture types, during fruit development and ripening. The firmness and pectin content of the fruits rapidly decreased and were much lower in ‘Fuji’ than in ‘Qinguan’ from 105 days after full bloom (DAFB). Transmission electron microscopy showed that the pectin-rich middle lamella was substantially degraded from 105 to 180 DAFB in the two apple cultivars. However, the degradation was more severe in ‘Fuji’ than in ‘Qinguan.’ Subcellular localization analysis showed that the Mdβ-Gal1, Mdβ-Gal2, and Mdβ-Gal5 proteins were located in the cell wall. β-Gal activity continuously increased during all fruit developmental stages and was much higher in the mature fruits of ‘Fuji’ than in those of ‘Qinguan,’ indicating that pectin was degraded by β-Gal. Consistent with the enzyme activities, expression levels of *β-Gal* genes (*Mdβ-Gal1*, *Mdβ-Gal2*, and *Mdβ-Gal5*) showed only slight changes from 60 to 105 DAFB but then dramatically increased until fruit ripening, with higher values in ‘Fuji’ than in ‘Qinguan.’ Furthermore, we found that activities of deletion derivatives in the *Mdβ-Gal2* promoter and transcript level of *Mdβ-Gal2* were induced by the treatment with methyl jasmonate (MeJA) and ethylene (ETH) hormones. Two ETH and one MeJA hormone-responsive elements were identified by analyzing the promoter sequence. These results suggest that β-Gals, induced by ETH and MeJA, are involved in different fruit texture types of apple cultivars by influencing the degradation of pectin during the mature fruit stage.

## Introduction

Fruit ripening is a complex and coordinated developmental process that involves a range of physiological, biochemical, and genetic events affecting the qualitative traits, such as the color, texture, and taste (Prasanna et al., 2007). Apple fruit texture, described by firmness, crispness, and juiciness, has been extensively studied because of consumer preferences (Peneau et al., 2006). Fruit texture is mainly affected by disassembly of the cell wall structure and depolymerization of cell wall components (Brummell and Harpster, 2001), which involve the coordinated and interdependent actions of cell wall hydrolytic enzymes, including polygalacturonase (PG), pectin methylesterase (PME), pectate lyase (PL), endo-1,4-β-D-glucanase (EGase), xyloglucan-endotransglycosylase (XET), β-galactosidase (β-Gal), α-L-arabinofuranosidase (α-AF), and expansion (EXP) (Goulao et al., 2007). Among these enzymes, PG has been extensively studied and was believed to be the most potent enzyme regulating fruit softening (Grierson and Tucker, 1983; Li et al., 2013). However, this has not been proven in transgenic tomatoes with suppressed PG expression (Giovannoni et al., 1989). The loss of galactan residues from the cell wall seems to play a role during apple fruit development and ripening (Ross et al., 1994; Pena and Carpita, 2004; Ng et al., 2015). In our previous study, we have found that the β-Gal activity showed a prominent difference among apple cultivars with different texture types during fruit ripening (Gao et al., 2016).

β-galactan is mainly present on side chains of the polysaccharide rhamnogalacturonan-I (Schols et al., 1995). These chains are entangled with glucan chains of cellulose (Zykwinska et al., 2007), forming a dense network that contributes to the extensibility, strength, and porosity of the cell wall (Ulvskov et al., 2005; Larsen et al., 2011). β-Gal (EC 3.2.1.23), a glycosidase, contains a consensus sequence of the putative active site, G–G–P–[LIVM]–x–Q–x–E–N–E–[FY], of glycosyl hydrolase family 35 (GH35) proteins (Henrissat, 1998). The role of β-Gal is to remove terminal, non-reducing β-D-galactosyl residues of hemicellulose and pectin from the cell wall (Smith and Gross, 2000). The β-Gal enzyme is believed to accelerate fruit softening by increasing the porosity of the cell wall and enhancing the access of other cell wall-degrading enzymes (Pena and Carpita, 2004; Ng et al., 2013, 2015).

β-Gals are typically encoded by members of large gene families and have been isolated from various plants, including *Arabidopsis* (Ahn et al., 2007), tomato (Smith and Gross, 2000), strawberry (Trainotti et al., 2001), pear (Tateishi et al., 2001), and papaya (do Prado et al., 2016). In mature tomatoes, the gene that is most abundantly transcribed is *Slβ-Gal4*, which belongs to one of the seven *β-Gal* gene families (Smith and Gross, 2000). The increase in β-Gal activity and the decrease in the cell wall galactosyl content in antisense *Slβ-Gal4* tomato lines suggested that this gene may be involved in cell wall modification, thus preventing fruit softening (Smith et al., 2002). Paniagua et al. (2016) have reached a similar conclusion regarding strawberries when they used antisense-mediated downregulation of *Faβ-Gal4*. A recent study has identified some selective sweeps underlying quantitative trait loci/genes of important fruit quality traits, including the fruit texture and flavor, and provided evidence supporting the contribution of *β-Gal* to the constant selection of cultivars with firm fruits in the history of apple domestication (Duan et al., 2017).

Methyl jasmonate (MeJA) is an important plant hormone in biotic and abiotic stress tolerance as well as in flowering and seed and fruit maturation (Saniewski et al., 1987; Lalel et al., 2003). In addition, application of MeJA has been shown to increase the activities of cell wall modifying enzymes (Concha et al., 2013; Wei et al., 2017). In apples, MeJA treatment was shown to markedly increase the ethylene (ETH) release and to accelerate fruit softening (Li et al., 2017). ETH also plays a major role in ripening and softening of climacteric fruits (Bapat et al., 2010). Previous studies have shown that ETH induces the expression of *PG*, *β-Gal*, *PL*, *PME*, *XET*, and *EGase* genes, resulting in the rapid softening of fruits (Nishiyama et al., 2007; Shen et al., 2017). Moreover, a recent study has shown that β-Gal activity is highly correlated with the ETH production in apples (Gwanpua et al., 2014), thereby emphasizing the role of hormones in β-Gal regulation during apple fruit softening. However, most of previous studies have focused on fruit softening, and the function of β-Gal during fruit development and ripening in apple cultivars with different texture types remains unclear.

Among two late cultivars selected for this study, ‘Fuji,’ a major apple cultivar, has a soft and crisp texture type, whereas ‘Qinguan’ is characterized by firmness and toughness of matured fruits. We hypothesized that β-Gal, induced by ETH and MeJA, played important roles in the two different texture types of apple cultivars by degrading pectin. In the present study, we measured the contents of cell wall components and observed the ultrastructure of the cell wall in fruits of these two apple cultivars. Additionally, we investigated the expression patterns of *Mdβ-Gal* family members and attempted to elucidate the roles of these genes in apple fruit development and ripening. We also isolated the *Mdβ-Gal2* promoter and then used it to express a β-glucuronidase (GUS) reporter gene in transgenic tobacco plants under hormonal treatment to study the role of the hormones in *Mdβ-Gal2* transcription. Our findings may help elucidate the potential role of β-Gal in apple texture, which is a critical fruit quality in the series of fruit evaluation indicators.

## Materials and Methods

### Plant Materials

‘Qinguan’ and ‘Fuji’ apple trees were planted in 2000 at Experimental Station of Northwest A&F University, Baishui County, Shaanxi Province, China. Fruits of ‘Fuji’ and ‘Qinguan’ were harvested at 180 days after full bloom (DAFB) determined by taste, ground color and degree of starch clearance. Uniform size, appearance and without external damage fruits, young leaves, function leaves, young stems and full flowers were selected from three trees at the same block. Fruits of ‘Qinguan’ and ‘Fuji’ were collected every 15 days from 60 DAFB until harvest. In addition, ‘Qinguan’ fruits were sprayed with 0.5 mM MeJA or 0.5 g⋅L^-1^ ETH at 165 DAFB, and these fruits were collected at harvest. Untreated fruits were used as control. Fruits were immediately transported to the laboratory at Northwest A&F University. Whole fruits were used for firmness tests, and the flesh tissues were cut into small pieces, pooled, immediately frozen in liquid nitrogen, and stored at -80°C for biochemical and molecular analyses.

Tobacco plants (*Nicotiana tabacum* cv. NC89) were cultured in an artificial climate incubator under a 16/8 h photoperiod, with a 25/20°C (day/night) temperature cycle and 70% relative humidity. Six- to eight-week-old, growth consistent plants were used for *Agrobacterium*-mediated transient assays.

### Firmness Measure

Fruit firmness was measured at three equidistant sites near the fruit equatorial axis of 15 peeled fruits using a texture analyzer (FTA GS-15, Germany; test depth, 8 mm) equipped with a 10 mm diameter flat probe. The maximum force formed during the test was recorded. Firmness was calculated as the average force.

### Cell Wall Material and β-Gal Activity Analysis

The cell wall extraction procedure used was a modified method from Melton and Smith (2001). Briefly, cell wall material (CWM) was extracted using Tris-phenol buffer and dimethyl sulfoxide (DMSO) from the frozen flesh (3.0 g). Pectin and hemicellulose polysaccharides were sequentially extracted using (1,2-cyclohhexylenedinitrilo)-tetraacetic acid (CDTA, 0.05 M), Na_2_CO_3_ (0.05 M), and KOH (1 and 4 M) from the obtained CWM. The final cell wall residue was mainly cellulose. The carbazole-ethanol method and anthrone-sulfuric acid method were applied to determine the contents of pectin and hemicellulose, which were expressed as mg⋅g^-1^ fresh weight (FW). Cell wall enzymes were extracted used the methods described by Brummell et al. (2004). The β-Gal enzyme activity was expressed as μmol PNP (p-nitrophenol) min^-1^⋅g^-1^FW by using p-nitrophenyl-β-D-galactopyranoside as substrate.

### Ultrastructural Analysis of Cell Wall

The ultrastructural analysis of the apple flesh cell wall by transmission electron microscopy (TEM) was performed as described previously (Tong et al., 1999) with modification. Small pieces of apple flesh (10 mm^3^) were cut, fixed in fixative solution (4% glutaraldehyde) at 4°C for 6 h, washed in phosphate-buffered saline (PBS, pH 6.8) at least three times, and postfixed in 1% osmium tetroxide for 2 h. After dehydration through a graded ethanol-water series, the samples were infiltrated, embedded, and polymerized in LR White resin (London Resin Company, Reading, United Kingtom) at 55°C for 48 h. Ultra-thin sections (approximately 70 nm thick) were cut with an ultramicrotome (Ultracut-R, Leica, Germany), and then analyzed with a transmission electron microscope (JEM 1230, JEOL, Japan).

### Bioinformatic Analysis

The nucleotide sequences of *Mdβ-Gal* genes were searched and confirmed from National Center for Biotechnology Information (NCBI), designated as *Mdβ-Gal1-13*. A phylogenetic tree was generated with MEGA 7.0 using the neighbor-joining method and 1000 bootstrap replicates with default parameters for deduced amino acid sequences of 13 apple *β-Gal* genes and 50 *β-Gal* homologs from other species. The alignment of the amino acid sequence was performed by DNAMAN, and conserved motifs within the apple β-Gal proteins were identified using the Conserved Domain Database^1^.

### RT-qPCR

RNA was extracted and purified using a Quick RNA isolation kit (Huayueyang, Beijing, China). Reverse transcription was performed to synthesize cDNA from 1 μg total RNA by random primers using the PrimeScript^TM^ RT reagent Kit with gDNA Eraser (TaKaRa, Kyoto, Japan). RT-qPCR was carried out with an iQ5 Multicolor Real-Time PCR Detection System (Bio-Rad, Hercules, CA, United States) using SYBR^®^ Green Master Mix (TaKaRa, Kyoto, Japan). The RT-qPCR program was as follows: 94°C for 30 s and then 40 cycles of 95°C for 5 s, 60°C for 30 s. The mRNA data was quantified by the 2^-ΔΔCt^ method (Livak and Schmittgen, 2001), using the *Actin* gene served as an internal control and the gene expression level in the stem tissue as a nominal value of 1. The specific primers used for RT-qPCR were designed and synthesized, and are shown in Supplementary Table S1.

### Subcellular Localization

The full *Mdβ-Gal1/2/16* coding sequences without the stop codons were amplified by PCR (primers are listed in Supplementary Table S1). The amplified fragments were digested by the appropriate restriction enzymes, which are underlined in the primers list (Supplementary Table S1), and were then ligated into the pCAMBIA1302-GFP vector, digested by the corresponding enzymes, resulting in Mdβ-Gal1/2/16-GFP vectors. The three fusion constructs and the control GFP vector were bombarded into onion epidermal cells by a Biolistic^®^ PDS-1000/He particle delivery system (Bio-Rad, Hercules, CA, United States). All bombarded onion epidermal cells were incubated on Murashige-Skoog medium for 24 h at 22°C in the dark and were then plasmolyzed in 30% sucrose for 10 min. GFP fluorescence was observed with a fluorescence microscopy (BX51; OLYMPUS, Japan). All bombard tests were repeated at least three times.

### Promoter Cloning and Bioinformatic Analysis

Genomic DNA was extracted from apple leaves using the Plant Genomic DNA Kit (Tiangen, Beijing, China). In order to obtain the promoter region of the *Mdβ-Gal2* gene, the primers, Mdβ-Gal2p1-F and Mdβ-Gal2p1-R, were used to amplify about 2 kb from ‘Qinguan’ genomic DNA between an upstream DNA sequence and a portion of the *Mdβ-Gal2* gene downstream of the transcriptional start site. Then, the second round of PCR was performed using Mdβ-Gal2p2-F and Mdβ-Gal2p2-R as primers (all primers are listed in Supplementary Table S1) and the first PCR product as a template. The final purified product was cloned into the pGEMT^®^-T Easy Vector (Promega, Madison, WI, United States) and sequenced at Aoke (Beijing, China). Sequence analyses were carried out using DNASTAR software. The putative *cis*-element of the *Mdβ-Gal2* promoter was identified using PLANTCARE^2^ (Lescot et al., 2002).

### Construction of Promoter-GUS (β-Glucuronidase) Fusion and Deletion Vectors

The full length of *Mdβ-Gal2* promoter (1494 bp) and a series of deletion fragment of the *Mdβ-Gal2* promoter (-1252 bp, -990 bp, -680 bp, and -397 bp from the translational start site of *Mdβ-Gal2*) were, respectively, amplified by PCR, adding a *Pst*I restriction site in all forward primers and a *Bam*HI site in all reverse primers. Further, the Cauliflower mosaic virus (CaMV) 35S promoter was amplified from pCAMBIA1302 by using primers containing the same restriction enzyme sites as mentioned above. All amplified regions were cloned upstream of the ATG in the GUS gene of the pCAMBIA1391z binary vector, following double-digestion with *Pst*I/*Bam*HI and ligation. These recombinants, which were positively identified by sequencing and were named as P1494, P1252, P990, P680, P397, and CaMV35S, were transformed into *Agrobacterium tumefaciens* (strain GV3101) via the freeze-thaw method. A schematic diagram of promoter deletion is illustrated in **Figure 5A**. The primers that were used are presented in Supplementary Table S1.

### *Agrobacterium*-Based Transient Assay and Hormone Treatment

The *Agrobacterium*-based transient assay was performed as described previously (Rothstein et al., 1987) with modification. The *Agrobacterium* strain, GV3101 (transformed with the appropriate construct) was streaked on LB agar with rifampicin (50 mg⋅L^-1^), kanamycin (50 mg⋅L^-1^) and gentamicin (50 mg⋅L^-1^) and grown at 28°C for 2 days. Colonies were selected and grown overnight in LB broth with the above mentioned antibiotics. *Agrobacterium* cultures were centrifuged, and then resuspended in infiltration solution (27.8 mM glucose, 100 μM acetosyringone, 50 mM 2-(N-morpholino) ethanesulfonic acid). The cultures were diluted until their OD_600_ reached 0.5 and were then infiltrated into tobacco leaves using a vacuum pump. The infiltrated leaves were maintained on a wet filter in a controlled-environment growth chamber under normal growth conditions. After 24 h, the leaves were, respectively, soaked in MeJA (1 mM) and ETH (1 g⋅L^-1^) solutions for 5 min, and then incubation continued for 24 h.

### GUS Histochemical and Fluorometric Assays

For the histochemical analysis of GUS, the infiltrated tobacco leaves were immersed in GUS staining solution (100 mM sodium phosphate buffer, pH 7.0, 0.5 mM K_3_Fe(CN)_6_, 0.5 mM K_4_Fe(CN)_6_, 10 mM Na_2_EDTA, 0.5 mg⋅mL^-1^ 5-bromo-4-chloro-3-indolyl β-D-glucuronide (X-GluC), 0.1% Triton X-100) for 12 h at 37°C in darkness and were then detained in multiple changes of 75% ethanol. Further, GUS activity was expressed as nmol 4-methylumbelliferone (4-MU, Sigma-Aldrich) generated from the corresponding glucuronide (4-methylumbelliferyl β-D-glucuronide, MUG) per minute per milligram of soluble protein (Jefferson, 1987). To measure this activity, the 4-MU product was quantified by fluorescence intensity with a Hitachi 850 Fluorescence Spectrophotometer (Hitachi, Tokyo, Japan). The protein concentration was determined by the method of Bradford (Bradford, 1976).

### Statistical Analysis

All data were obtained from at least three independent experiments. Values were expressed as mean ± standard error of the mean. The data were tested through analysis of variance using SPSS statistics v19.0 software, and the means were compared with student’s *t*-test. Difference between groups was considered significantly different if *p* < 0.05.

## Results

### Physiological Characterization of Apple Fruits During Development and Ripening

**FIGURE 1:**
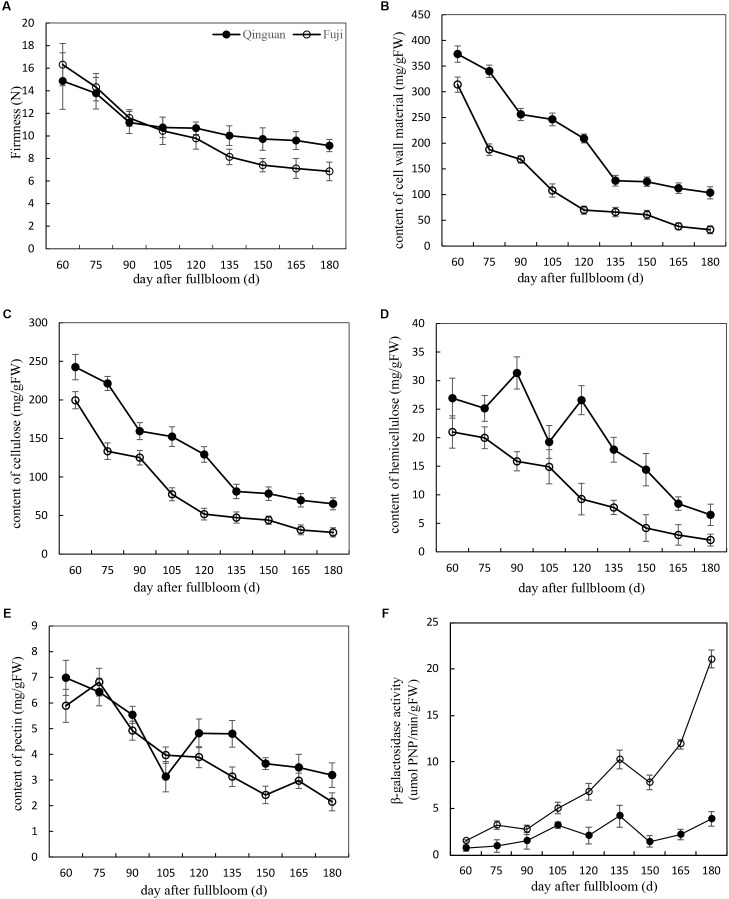
The variation of physiological characters in ‘Fuji’ and ‘Qinguan’ apple during fruit development and ripening. **(A)** Fruit firmness, **(B)** the content of cell wall material, **(C)** cellulose, **(D)** hemicellulose, **(E)** pectin and **(F)** β-galactosidase activity. Data are presented as means from three independent experiments, the *vertical bars* indicate the standard error of the mean.

Fruit firmness, CWMs, and β-Gal activity were measured in ‘Fuji’ and ‘Qinguan’ fruits at 15-day intervals from 60 DAFB. The flesh firmness rapidly decreased during the fruitlet stage (before 90 DAFB) and then slowly decreased during the expanding fruit stage (from 90 to 150 DAFB) and mature fruit stage (from 150 to 180 DAFB). The degree of decline was different between the cultivars, resulting in a difference in the final fruit firmness. Compared with that at the fruitlet stage, the firmness of ‘Fuji’ at the mature fruit stage decreased by 67%, whereas that of ‘Qinguan’ was slower to decrease, resulting in a 56% reduction. During the fruitlet and early expanding fruit stages, the fruit firmness was higher in ‘Fuji’ than in ‘Qinguan,’ but during the late expanding and mature fruit stages, the trend was reversed (**Figure 1A**).

The contents of CWMs, cellulose, and hemicellulose in the two apple cultivars showed similar decreasing trends through all developmental stages. The decrease was rapid from the fruitlet stage to the expanding fruit stage, with a slight change during the mature fruit stage (**Figures 1B–D**). The contents were higher in ‘Qinguan’ than in ‘Fuji,’ which was consistent with the firmness observed during the expanding and mature fruit stages but contrasted with that in the fruitlet stage.

The pectin content in the two apple cultivars was only slightly different at the fruitlet stage but showed distinct differences during the expanding and mature fruit stages (**Figure 1E**), along with corresponding firmness changes in the two apple cultivars.

The activities of β-Gal in the two apple cultivars showed almost no changes in the fruitlet stage but were significantly different in the expanding and mature fruit stages. Overall, the activity of β-Gal in ‘Qinguan’ remained stable. However, in ‘Fuji,’ the activity of β-Gal progressively increased, and the rate of the increase was significantly accelerated at the mature fruit stage. Furthermore, in mature fruits of ‘Fuji,’ the activity of β-Gal was 7.75-fold higher than that in ‘Qinguan’ (**Figure 1F**). In summary, pectin degradation by β-Gal may affect the fruit texture of apple cultivars.

### Ultrastructural Analysis of Apple Fruits at Different Stages

**FIGURE 2:**
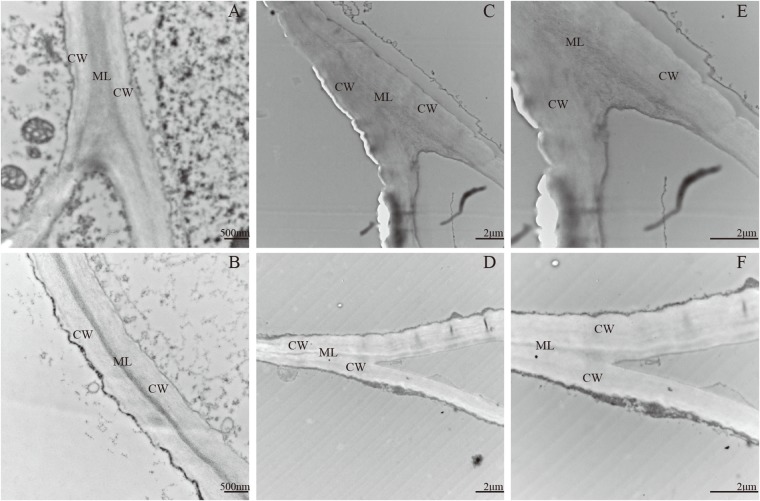
Transmission electron microscope of two apple cultivars during fruit development and ripening. **(A,C,E)** Fuji; **(B,D,F)** Qinguan; **(A,B)** 105 days after full bloom; **(C,D)** 180 days after full bloom. CW: cell wall, ML: middle lamella. **(A,B)**: 20000 ×, size bars, 500 nm; **(C,D)**: 6000 ×, size bars, 2 μm.

Pectin, the main component of the middle lamella, affects the cell wall loosening and cell adhesion and shows difference between the two apple cultivars. Ultrastructural changes in cell walls were determined by TEM during the fruitlet and mature fruit stages in ‘Qinguan’ and ‘Fuji’ (**Figure 2**). In the fruitlet stage, adjacent regions of cell walls in the two cultivars appeared as broad light–dark–light bands. The cell wall structure showed an orderly arrangement, with tightly packed fibrous material and a conspicuous middle lamella (**Figures 2A,B**). Ripening fruits showed a severely reduced dark region and damaged cell wall integrity. The broad light and dark bands nearly disappeared and were replaced by thin lines or cumulate laminates. The electron-dense cell wall became electron-lucent, particularly at cell corners. Differences were observed between the two cultivars in that the region previously occupied by the middle lamella was more obscure, particularly at tricellular junctions, and the fibrous material from the cell wall was more degraded and appeared to be dispersed in ‘Fuji’ (**Figures 2C,D**). These results indicated that the differences in the pectin content were the primary reason for the different texture types of apple cultivars.

### Phylogenetic Analysis of Mdβ-Gal Proteins

**FIGURE 3:**
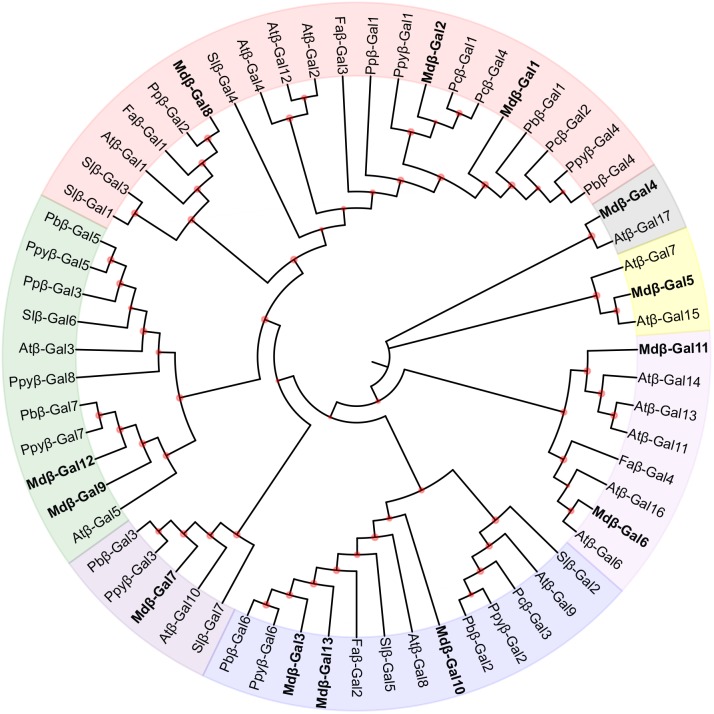
Phylogenetic tree of β-Gals deduced amino acid from *Malus domestica* with other species. Species shown are *Arabidopsis thaliana* (Atβ-Gal), *Fragaria ananassa* (Faβ-Gal), *Malus domestica* (Mdβ-Gal), *Pyrus bretschneideri* (Pbβ-Gal), *Pyrus communis* (Pcβ-Gal), *Prunus persica* (Ppβ-Gal), *Pyrus pyrifolia* (Ppyβ-Gal), and *Solanum lycopersicum* (Slβ-Gal). The phylogenetic tree was drawn using MEGA 7.0 software by the neighbor-joining method with 1000 replications. The reliabilities of internal branches are shown by the orange nodes. which (< 50%) were hidden. β-Gals from *Malus domestica* were marker in bold as shown in each subgroup. The GeneBank accession numbers used to build the tree were listed (Supplementary Table S3).

The β-Gal enzymes are encoded by a family of *Mdβ-Gal* genes. To determine the evolutionary relationship of the *β-Gal*s among plant species, a phylogenetic tree was created using full-length β-Gal protein sequences from apple, tomato, and other selected fruit species (**Figure 3**). Thirteen Mdβ-Gal proteins were clustered into seven subgroups (A–G). Subgroup A included Mdβ-Gal1, Mdβ-Gal2, and Mdβ-Gal8, together with Slβ-Gal4, which has previously been confirmed to affect fruit softening, and Faβ-Gal1, which has shown increased expression during fruit ripening, reaching a maximum in red fruits. Mdβ-Gal4, which lacked the signal peptide, was classified in subgroup B, together with Atβ-Gal17. Mdβ-Gal5, Mdβ-Gal6, and Mdβ-Gal11 were classified in subgroup C/D, together with Atβ-Gal8, which was expressed mostly in flowers. Additionally, Mdβ-Gal3, Mdβ-Gal7, Mdβ-Gal10, and Mdβ-Gal13 were classified in subgroup E/F, together with Pcβ-Gal3, which was highly expressed in early stages of fruit development and decreased toward fruit maturity. Mdβ-Gal9 and Mdβ-Gal12 belonged to subgroup G.

All the Mdβ-Gal proteins contained a putative consensus sequence, G–G–P–[LIVM](2)–x(2)–Q–x–E–N–E–[FY], belonging to GH35 proteins, and all, exception Mdβ-Gal4, contained a predicted signal peptide. Ten Mdβ-Gal proteins also possessed a Gal lectin domain at their C-terminus, which contributes to the substrate specificity of β-Gals. Mdβ-Gal5 carried an additional β-Gal jelly roll domain between the GH35 and Gal_lectin domains, whereas Mdβ-Gal11 and Mdβ-Gal12 carried two GH2N domains between the GH35 and Gal_lectin domains (Supplementary Figure S1). The functions of these extra domains remain unclear.

### Expression of *Mdβ-Gal* Genes During Fruit Development and Ripening

**FIGURE 4:**
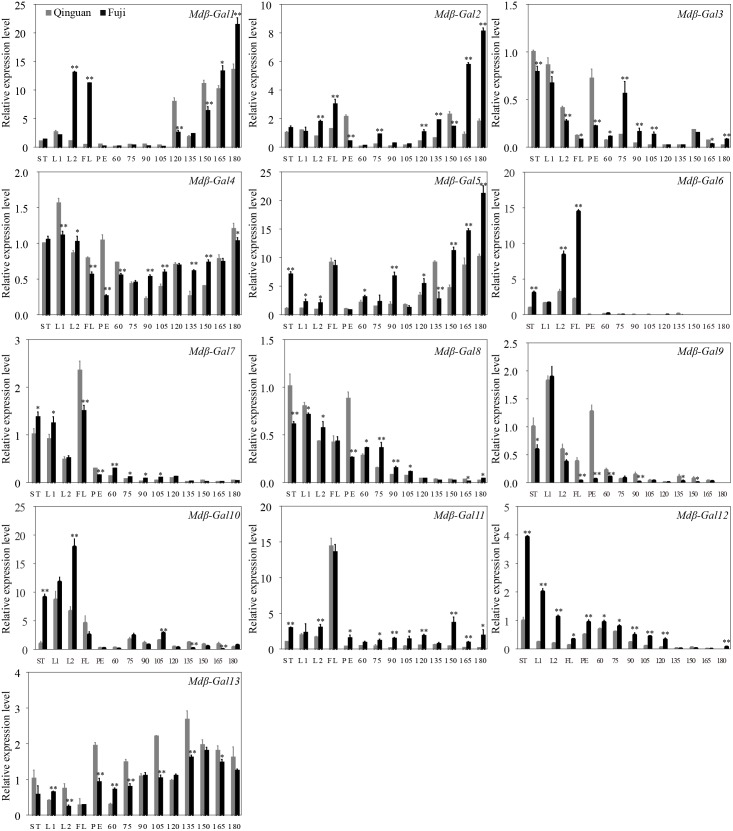
The expression level of *Mdβ-Gal* genes in various tissues and developing phases on apple fruits of ‘Qinguan’ and ‘Fuji,’ respectively. The *Mdβ-Gal* mRNA levels are relative to those of *Actin* mRNA. Significant difference analysis between two cultivars at the same time-point is indicated by student’s *t*-test probabilities: ^∗^*p* < 0.05; ^∗∗^*p* < 0.01. Mean values and standard error of the mean from three independent experimental series are shown. ST: stem; L1: young leaf; L2: function leaf; FL: flower; PE: fruit peel; 60, 75, 90, 105, 120, 135, 150, 165, 180: days after full bloom.

The transcription patterns of the 13 *Mdβ-Gal* genes were assessed in different apple tissues and developmental stages by RT-qPCR and were compared between the two apple cultivars (**Figure 4**). The results indicated that *Mdβ-Gal4* and *Mdβ-Gal13* were expressed at constant levels in all tissues. The other genes showed tissue-specific expression patterns. Five *Mdβ-Gal* genes (*Mdβ-Gal3*, *Mdβ-Gal8*, *Mdβ-Gal9*, *Mdβ-Gal10*, and *Mdβ-Gal12*) showed little expression in fruits. *Mdβ-Gal6*, *Mdβ-Gal7*, and *Mdβ-Gal11* were expressed mostly in flowers. *Mdβ-Gal1*, *Mdβ-Gal2*, and *Mdβ-Gal5* were most highly expressed in reproductive organs, such as flowers and fruits.

At different fruit developmental stages, the *Mdβ-Gal* genes showed three different expression patterns as follows: (1) the expression levels of *Mdβ-Gal1*, *Mdβ-Gal2*, *Mdβ-Gal3*, *Mdβ-Gal5*, and *Mdβ-Gal11* gradually increased during fruit development and were higher in ‘Fuji’ than in ‘Qinguan’; (2) the expression levels of *Mdβ-Gal6*, *Mdβ-Gal7*, *Mdβ-Gal8*, *Mdβ-Gal9*, and *Mdβ-Gal12* continuously decreased during fruit development or were low; and (3) the expression levels of *Mdβ-Gal10* and *Mdβ-Gal13* gradually increased during fruit development but exhibited a rapid decrease at later developmental stages. Taken together, these findings suggested that *Mdβ-Gal1*, *Mdβ-Gal2*, and *Mdβ-Gal5* might affect the fruit texture types of apple cultivars.

### Subcellular Localization

To elucidate the roles of Mdβ-Gal1, Mdβ-Gal2, and Mdβ-Gal5, four different constructs, encoding GFP fusion proteins (Mdβ-Gal1–GFP, Mdβ-Gal2–GFP, and Mdβ-Gal5–GFP, as well as the GFP control) were transfected into onion cells. After incubation for 24 h, the cells transfected with the GFP control showed the GFP localization in protoplasts and cell walls (Supplementary Figures S2K–M). In contrast, the Mdβ-Gal1, Mdβ-Gal2, and Mdβ-Gal5 fusion proteins were detected only in the cell walls of plasmolyzed cells (Supplementary Figures S2A–J). These results indicated that the Mdβ-Gal1, Mdβ-Gal2, and Mdβ-Gal5 proteins were targeted to the cell wall, which provided further evidence that their encoding genes were involved in the regulation of apple texture.

### Isolation and Sequence Analysis of the *Mdβ-Gal2* Promoter

To explore the regulation of *Mdβ-Gal2*, a 1494 bp 5′ flanking region, designated *pMdβ-Gal2*, was isolated from ‘Qinguan’ leaves and analyzed for putative *cis*-regulatory elements. The *pMdβ-Gal2* sequence and putative plant regulatory elements are shown in Supplementary Figure S3. The *cis*-regulatory elements in the promoter were classified into the following four functional groups (Supplementary Table S2): abiotic stress-, biotic stress-, light response-, and hormone response-related elements. Heat stress-responsive elements are an important type of abiotic stress-responsive elements. The biotic stress-responsive elements consisted of anaerobic-responsive elements, which are involved in the regulation of zein metabolism (O_2_ site), and a fungal elicitor-responsive element (Box-W1). The light-responsive elements consisted of a G-box and other typical elements, including an ABRE box, Box I, a GAG motif, and an Sp1 element. The hormone-responsive elements included a MeJA-responsive element (TGACG motif) and two ETH-responsive elements (EREs). The presence of these putative *cis*-regulatory elements indicated that *Mdβ-Gal2* may be partially involved in responses to hormones.

### Responsiveness of the *Mdβ-Gal2* to Hormonal Stress

**FIGURE 5:**
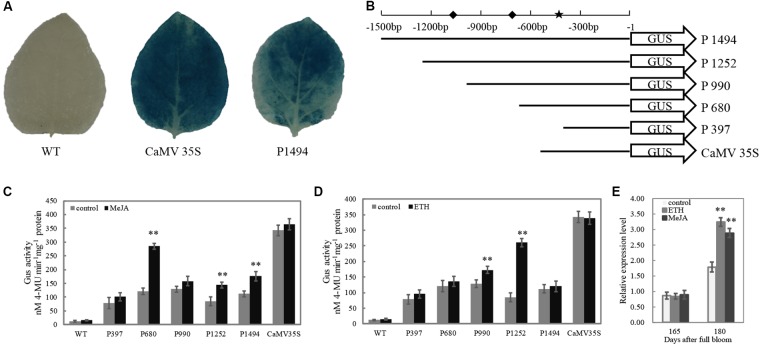
The activity of *Mdβ-Gal2* promoter in response to hormonal treatments. **(A)** Histochemical staining of transiently transformed tobacco leaves; **(B)** schematic diagram of vector constructs for the Mdβ-Gal2 promoter, 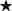: MeJA element; 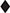: ETH element; **(C)** soaked in 1mM MeJA for 5 min; **(D)** soaked in 1 g⋅L^-1^ ETH for 5 min; **(E)** sprayed by 0.5 mM MeJA and 0.5 g⋅L^-1^ ETH. *Vertical bars* represent the standard error of the mean. Asterisks indicated statistically significant differences as determined by Student’s *t*-test (*^∗^p* < 0.05*; ^∗∗^p* < 0.01).

To test the activity of the *Mdβ-Gal2* promoter, the promoter–GUS reporter construct (P1494) was analyzed in an *Agrobacterium*-mediated transient expression system. The CaMV35S–GUS (CaMV35S) construct was used as the positive control. No GUS activity was observed in the wild-type strain. A histochemical assay confirmed that the 1494 bp *Mdβ-Gal2* promoter was able to drive the expression of the GUS reporter gene (**Figure 5A**), although the promoter activity of *Mdβ-Gal2* was much lower than that of the positive control.

To elucidate whether the differential gene expression patterns of *Mdβ-Gal2* in the two apple cultivars were correlated with the regulatory elements in its promoter, we prepared a series of *pMdβ-Gal2* deletions and fused the clones with the GUS reporter gene (**Figure 5B**). Compared with that of the control, the GUS activity of pMdβ-Gal2–GUS was substantially increased by MeJA and ETH treatments, by approximately 2.02- and 1.62-fold, respectively. Significant MeJA-inducible promoter activity was detected in tobacco leaves harboring the P1494, P680, and P397 constructs (**Figure 5C**). Additionally, significant ETH-inducible promoter activity was detected in the transformants with the P1252 and P990 constructs (**Figure 5D**). In all of the treatments, wild-type leaves and those transformed with the positive control construct showed no obvious inducible GUS activity. In addition, the transcript level of *Mdβ-Gal2* increased by the pre-harvest application of MeJA and ETH at harvest (**Figure 5E**). These results indicated that the *Mdβ-Gal2* was induced by hormonal stress.

## Discussion

The apple cultivars ‘Fuji’ and ‘Qinguan’ show different fruit texture during development and ripening. The firmness of ‘Fuji’ apples decreased rapidly from an initial value of 16.32 N in the fruitlet stage to 6.86 N in the mature stage, whereas that of ‘Qinguan’ showed a slower decrease during fruit development and ripening. β-Gal, a pectin enzyme, plays an important role in fruit ripening and softening (Ng et al., 2015; Dheilly et al., 2016). In our study, we explored the regulatory mechanisms of fruit texture by β-Gal in two different apple cultivars.

### Cell Wall Composition and Ultrastructure and β-Gal Enzyme in Different Apple Cultivars

Texture changes in fruits are a consequence of the degradation of cell wall polysaccharides, including cellulose, hemicellulose, and pectin (Forster et al., 2002). Cybulska et al. (2013) and Chen et al. (2009) have reported that thicker cellulose and hemicellulose layers were associated with desirable texture properties, whereas Zhang et al. (2012) have demonstrated that long and linear single pectin chains were detached during peach storage. In our study, we found that the contents of cellulose and hemicellulose were higher in ‘Qinguan’ than in ‘Fuji’ (**Figures 1C,D**), which was consistent with the observed firmness in the expanding and mature fruit stages but not with that in the fruitlet stage. However, the pectin content was markedly different between the two cultivars, consistent with the apple firmness at all stages (**Figures 1A,E**). Thus, we concluded that the pectin content is the main contributor to the differences in fruit texture between the two apple cultivars. This hypothesis was confirmed by TEM observations during the fruitlet and mature stages (**Figure 2**). In the fruitlet stage, the middle lamella was clear, and the cell wall network was dense. In contrast, in the mature fruit stage, the middle lamella was almost invisible, and the cell wall structure became loose. Moreover, the region of the middle lamella was more dispersed in ‘Fuji’.

Pectin, the main component of the middle lamella, is degraded by a series of enzymes, including PG, PL, PME, β-Gal, and α-AF (Jarvis et al., 2003; Ng et al., 2013). β-Gal and α-AF affect the storability of apples more than PG and PME do (Wei et al., 2010). Pena and Carpita (2004) have suggested that the loss of galactan occurs during the growth and maturation phase, whereas the loss of highly branched arabinans occurs in storage. In our study, β-Gal levels were the lowest in the fruitlet stage and then increased until the mature fruit stage. The activity in ‘Fuji’ was higher than that in ‘Qinguan’ at all developmental stages, and the difference became increasingly significant from the fruit expanding stage (**Figure 1F**). These changes were consistent with the results of Dheilly et al. (2016) and Gwanpua et al. (2016), implying that alterations in the β-Gal activity lead to differences in pectin solubilization and the cell wall structure, and result in different texture types. Other investigators have shown that β-Gal activity decreases from the fruitlet to harvest or peaks at the expanding fruit stage and then decreases until harvest (Goulao et al., 2007; Ng et al., 2015). This discrepancy may be due to variations among apple cultivars and differences in the expression levels of multiple enzyme isoforms during ripening.

### Texture-Associated Expression Patterns of *β-Gal* Genes in Different Apple Cultivars

To date, the roles of β-Gals have been investigated in many species (Smith and Gross, 2000; Tateishi et al., 2001; Ahn et al., 2007). In apples, molecular characterization of β-Gals has focused on changes in gene expression and responses to ETH. Gwanpua et al. (2016) have indicated that the expression levels of *Mdβ-Gal1* (MDP0000416548) and *Mdβ-Gal2* (MDP0000127542) significantly increased during fruit softening and were suppressed by 1-methylcyclopropene treatment. Ireland et al. (2014) have also demonstrated that ETH treatment of apple fruits resulted in an increased expression of *Mdβ-Gal2*. However, these studies were performed using stored apples, and thus these features have not been assessed in growing fruits.

In our study, expression profiles of 13 *Mdβ-Gal* genes were investigated in different tissues and stages during fruit development in two apple cultivars. Three *Mdβ-Gal* genes, *Mdβ-Gal1*, *Mdβ-Gal2*, and *Mdβ-Gal5*, were identified to be highly linked to the fruit ripening process. Moreover, ‘Fuji’ displayed higher expression levels of these genes than ‘Qinguan’ did (**Figure 4**), consistent with the β-Gal activities in the two apple cultivars (**Figure 1F**). These three proteins were also found to be located in the cell wall (Supplementary Figure S2), providing additional evidence that these genes are involved in the regulation of apple texture. A phylogenetic tree analysis showed that Mdβ-Gal1 and Mdβ-Gal2 were closely related to Ppyβ-Gal1 and Ppyβ-Gal4 (**Figure 3**), which are encoded by genes, known to act as ripening-specific genes in Japanese pears (Tateishi et al., 2005). The data suggest that these genes may play similar roles in apple and pear ripening. *Mdβ-Gal5*, a novel gene, was closely related to *Atβ-Gal7*. Ahn et al. (2007) have indicated that *Atβ-Gal7* was mostly expressed in flowers. In our study, *Mdβ-Gal5* showed high expression levels in flowers as well as in fruits during a late developmental stage (**Figure 4**), indicating that this gene may be involved in the regulation of fruit texture. Furthermore, Mdβ-Gal2 exhibited homology with the N-terminal amino acid sequence of β-Gal protein, except for two amino acids. The β-Gal protein was originally purified from ‘Granny Smith’ apple fruits by Ross et al. (1994) and shown to have a molecular mass of 78.5 kDa, which is somewhat lower than that of Mdβ-Gal2 (81.0 kDa), as predicted based on the nucleic acid sequence. Overall, these results demonstrated that *Mdβ-Gal2*, as a vital gene, may regulate the apple fruit texture during fruit ripening. Therefore, it will be important to further elucidate the regulatory mechanisms of this process by *Mdβ-Gal2*.

### Regulation of *Mdβ-Gal2* by Hormone Treatment

Promoters in plants play central roles in the temporal and spatial regulation of gene expression via specific *cis*-regulatory elements (Hernandez-Garcia and Finer, 2014). The pre-harvest spray application of MeJA and ETH at 165 DAFB resulted in significantly higher expression level of *Mdβ-Gal2* at harvest (**Figure 5E**). To elucidate the transcriptional regulation of *Mdβ-Gal2*, which may be useful for analyzing the gene function, the promoter of *Mdβ-Gal2* was isolated and functionally characterized. Previous studies have demonstrated that MeJA alters the expression levels of the *EG1* and *XTH1* genes in *Fragaria chiloensis* via MeJA-responsive elements in their promoter regions (Concha et al., 2013; Opazo et al., 2013). Similarly, the banana *EXP* gene is also regulated by MeJA (Ghosh et al., 2012). A histochemical GUS assay using transgenic tobacco plants suggested that the *pMdβ-Gal2* contained all the *cis*-acting regulatory elements required for the regulation of β-Gal (**Figure 5A**). For regulatory elements analysis, we identified a TGACG motif in the *Mdβ-Gal2* promoter sequence between positions -680 and -397 (**Figure 5B**), which corresponds to a *cis*-acting element involved in MeJA-responsiveness (Fink et al., 1988). We found that the activities of derivatives with deletions in the *Mdβ-Gal2* promoter, containing the TGACG motif, were strongly induced in tobacco leaves treated with MeJA (**Figure 5C**). Furthermore, we identified two EREs in the *Mdβ-Gal2* promoter region (**Figure 5B**). ETH, as the most important fruit ripening-related hormone, also regulated the activity of the *Mdβ-Gal2* promoter (**Figure 5D**). Taken together, our results demonstrated that the transcription of the *β-Gal* gene may be induced by MeJA and ETH via promoter activity, in which the TGACG motif and ERE act as important recognition sites.

## Conclusion

A broad analysis of β-Gal was performed to reveal enzyme activity, gene expression patterns, and hormone response during fruit development and ripening in two apple cultivars. Our results suggest that β-Gals, induced by ETH and MeJA, are involved in different fruit texture types of apple cultivars by influencing the degradation of pectin during the mature fruit stage.

## Author Contributions

HY performed the experiments, analyzed the data, and prepared the manuscript. JL, MD, BZ, and HL contributed to preparation of the materials, sample collection, and data analysis. RM, DQ, and YY contributed to analysis and discussion of the results and preparation of the manuscript. ZZ designed the experiments, discussed the data, and drafted the manuscript. All authors reviewed and approved the final manuscript.

## Conflict of Interest Statement

The authors declare that the research was conducted in the absence of any commercial or financial relationships that could be construed as a potential conflict of interest.

## Footnotes

1 https://www.ncbi.nlm.nih.gov/cdd

2 http://bioinformatics.psb.ugent.be/webtools/plantcare/html/
