# Supplementary material for: Analysis of β-Galactosidase During Fruit Development and Ripening in Two Different Texture Types of Apple Cultivars
Source: Front Plant Sci. 2018 Apr 24;9:539. doi: 10.3389/fpls.2018.00539 (PMC5928752; doi:10.3389/fpls.2018.00539)
Supplement: Supplementary file 1 [file Data_Sheet_1.DOCX]

Supplementary Material

Analysis of β-galactosidase during Fruit Development and Ripening in Two Different Texture Types of Apple Cultivars

Huijuan Yang, Junling Liu, Meile Dang, Bo Zhang, Hongguang Li, Rui Meng, Dong Qu, Yazhou Yang, Zhengyang Zhao*

*** Correspondence:** Zhengyang Zhao: zhaozy@nwsuaf.edu.cn

# Supplementary Figures and Tables

## Supplementary Figures


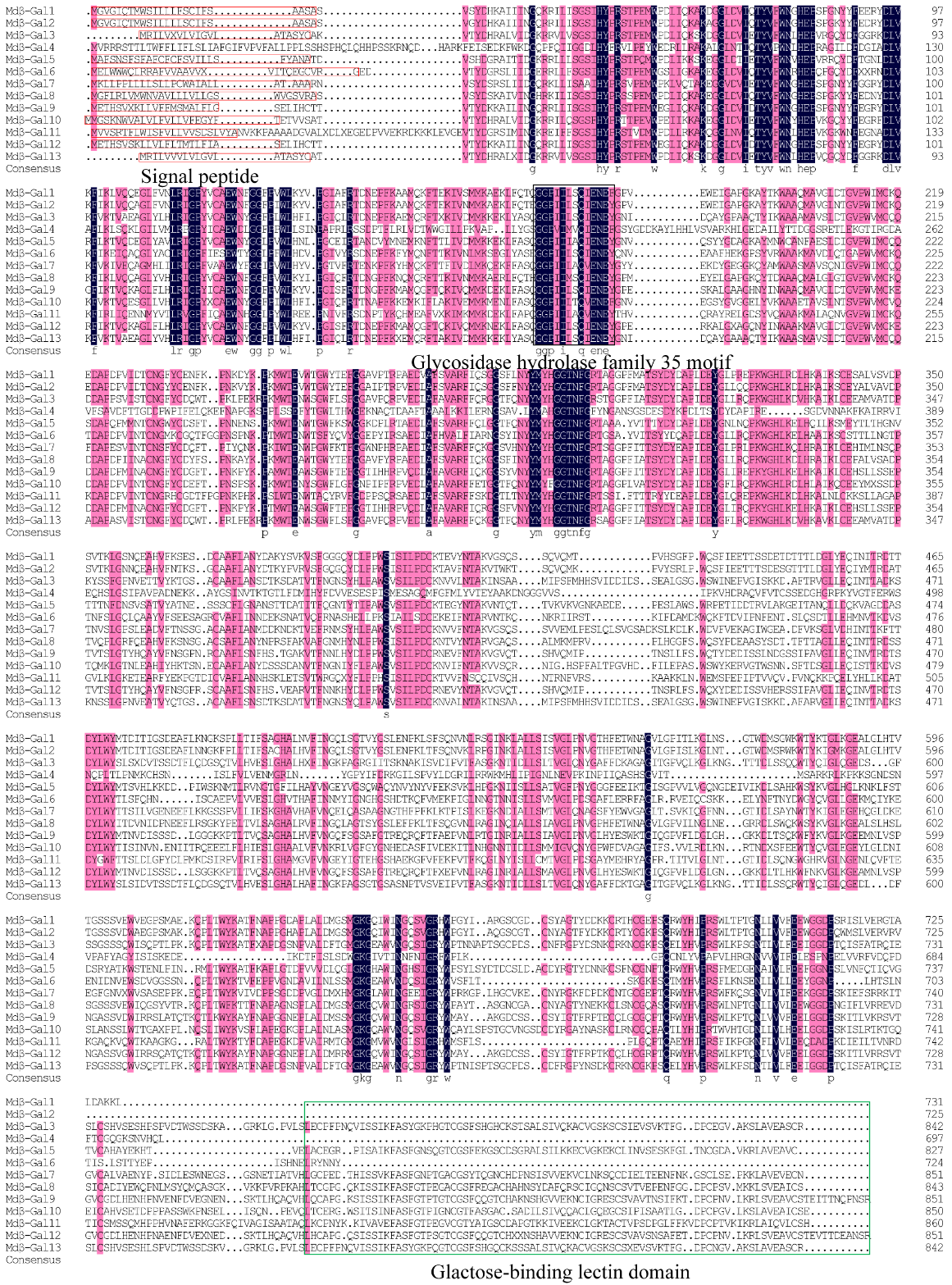


**Figure S1.** **The analysis of multiple Mdβ-Gals amino acid sequence alignment.** Different shading colors indicate different sequence similarities (navy blue: 100%, magenta: 75%). The predicted signal peptides at the N-terminal are boxed in red. The blue box indicates the consensus sequence of putative glycoside hydrolase family 35 motif. The predicted galactose-binding lectin domain at the C-terminal is boxed in green.


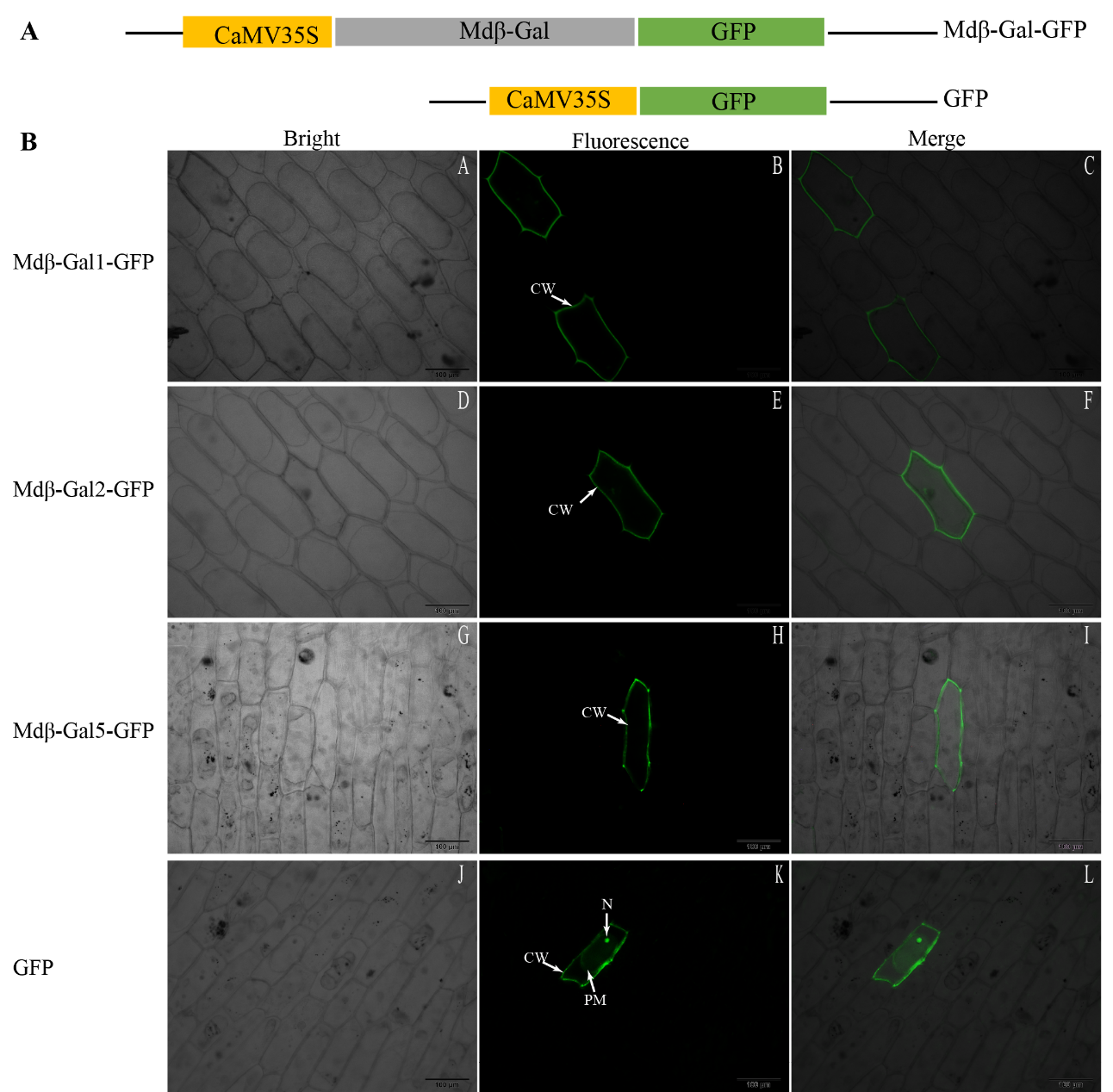


**Figure S2.** **Subcellular location patterns of Mdβ-Gals in onion epidermal cells.** (A) Diagram of the Mdβ-Gal-GFP fusion construct and only GFP construct as a negative control. (B) The images of Mdβ-Gal1-GFP, Mdβ-Gal2-GFP, Mdβ-Gal5-GFP and GFP control in transgenic onion cells, respectively shown by bright, fluorescence and merged field. Plasmolysis was induced with 30% sucrose. CW, cell wall; N, nucleus; PM, plasma membrane. Scale bar, 500μm.


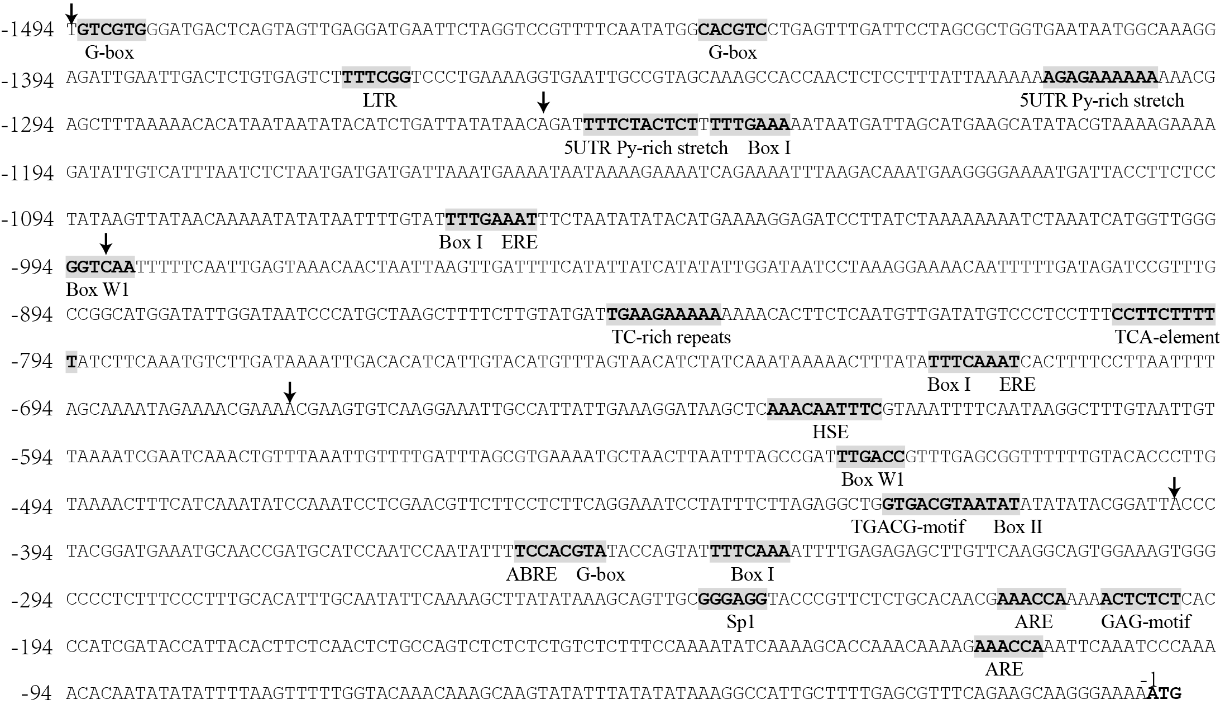


**Figure S3.** **The promoter nucleotide sequence analysis of the Mdβ-Gal2.** Mdβ-Gal2 translational start codon ATG (bold and boxed) numbered as ±1. Putative regulatory elements are shown in bold and shaded in grey. Names are given below the elements. Arrowheads represent the starting point of 5'-deleted derivatives.

# Supplementary Tables

### Table S1. Primer sequences used in this study

| Primer name | Primer sequences(5’-3’) | Purpose |
| --- | --- | --- |
| Mdβ-Gal1 | F: GGGAGTTCTTCTGTTGAATGGG | qRT-PCR |
|  | R: TTCCGGCATAAGAACAATCG |  |
| Mdβ-Gal2 | F: CGAAATACCCTGTTAGAGTGAGC |  |
|  | R: CGATGAATGACTGCCAAGGAA |  |
| Mdβ-Gal3 | F: TCAGAGGACCTTATGACTCCAACA |  |
|  | R: TTCCCGCTTTTGAATCTGAAC |  |
| Mdβ-Gal4 | F: GCCCGACCTCACTTCCTATG |  |
|  | R :GCTCTATGACCCTCCTAATTGCTT |  |
| Mdβ-Gal5 | F: TCTCAATGGGCTCAATACAACG |  |
|  | R: ATCACCATTTTGTCCCACCAG |  |
| Mdβ-Gal6 | F: AGGCATAGTTTACCGCTCAGA |  |
|  | R: TATTCATTCTCAATCTGCGACA |  |
| Mdβ-Gal7 | F: CTATGAAAAGGATTACGGAGAAGG |  |
|  | R: TTCTGATAGATAGGCGTAAACTGG |  |
| Mdβ-Gal8 | F: GAAAGTATCCTGAACTCACCGTCT |  |
|  | R: TGTGGACCAACATTCGGGAG |  |
| Mdβ-Gal9 | F: CACCGGGGAAGTCTATCTCG |  |
|  | R: CAACCGAGCAACTCTCACGC |  |
| Mdβ-Gal10 | F: GGGACATCTGCGTGACTTG |  |
|  | R: TCTTTGTGAGACAACCTTAGCG |  |
| Mdβ-Gal11 | F: CAATGGGCGTATTCGTCAA |  |
|  | R: CTGGGAGTCCCACTGTCATG |  |
| Mdβ-Gal12 | F: ACCACCCGAACGCTGAAAAT |  |
|  | R: CAACTGAGCAACTCTCGCGG |  |
| Mdβ-Gal13 | F: TTGAATGTTAAAACGGGAGCA |  |
|  | R: CAATACGAGCAGCTTTCCACA |  |
| Actin | F: TGTGTTTCCTAGTATTGTTGGTCGC |  |
|  | R: GCCAGATCTTCTCCATGTCATCC |  |
| Mdβ-Gal1full | F: CGGGATCCATGGGTGTTGGAATTCAAAC | β-Gal1 CDS clone |
|  | R: GGACTAGTGACTCTTACTCTTTCGACTA |  |
| Mdβ-Gal2full | F: CGGGATCCATGGGTGTTGGAATTCAAAC | β-Gal2 CDS clone |
|  | R: GGACTAGTGAGCTTCTTCGCGTCGAG |  |
| Mdβ-Gal16full | F: CGGGATCCATGGCTTTTTCCAACTCTTT | β-Gal16 CDS clone |
|  | R: GGACTAGTGCAAACAGCTTCCACAGCCA |  |
| Mdβ-Gal2p1 | F: CCCTCAAACTTAACCACCTGG | β-Gal2 promoter clone |
|  | R: GCTTCTGGGATAGTGAATGGAG |  |
| Mdβ-Gal2p2 | F: TGTCGTGGGATGACTCAGTAG |  |
|  | R: CATTTTTCCCTTGCTTCTGA |  |
| p1494 | F: GACTGCAGTGTCGTGGGATGACTCAGTAG | β-Gal2 full-length promoter clone |
| p1494 | R: CGGGATCCCATTTTTCCCTTGCTTCTGA |  |
| P397 | F: GACTGCAGCCCTACGGATGAAATGCAAC | β-Gal2 promoter deletion derivatives clone |
| P680 | F: GACTGCAGCGAAAACGAAGTGTCAAGGA |  |
| P990 | F: GACTGCAGAATTTTTCAATTGAGTAAAC |  |
| P1252 | F: GACTGCAGGATTTTCTACTCTTTTTGAA |  |

Letter ‘F’ and ‘R’ indicate the forward and reverse primers, respectively. Double underlines indicate the restriction enzyme site added in primers.

### Table S2. Cis-acting regulatory elements analysis of *Mdβ-Gal2* promoter sequence

| Regulatory element | Amount | Sequence | Function |
| --- | --- | --- | --- |
| 5UTR Py-rich stretch | 2 | TTTCTTCTCT | cis-acting element conferring high transcription levels |
| ABRE | 1 | ACGTGGA | cis-acting element involved in light responsiveness |
| ACE | 1 | TGGTTT | cis-acting regulatory element essential for the anaerobic induction |
| ARE | 2 | TGGTTT | cis-acting regulatory element essential for the anaerobic induction |
| Box I | 4 | TTTCAAA | light responsive element |
| Box II | 1 | GTGAGGTAATAT | part of a light responsive element |
| Box-W1 | 2 | TTGACC | fungal elicitor responsive element |
| CAAT-box | 45 | CAAT | common cis-acting element in promoter and enhancer regions |
| TGACG-motif | 1 | TGACG | cis-acting regulatory element involved in the MeJA-responsiveness |
| ERE | 2 | ATTTCAAA | ethylene-responsive element |
| G-Box | 3 | CACGTA | cis-acting regulatory element involved in light responsiveness |
| GAG-motif | 1 | AGAGAGT | part of a light responsive element |
| GATA-motif | 1 | AAGGATAAGG | part of a light responsive element |
| GCN4_motif | 2 | TGAGTCA | cis-regulatory element involved in endosperm expression |
| HSE | 1 | AAAAAATTTC | cis-acting element involved in heat stress responsiveness |
| I-box | 1 | AGATAAGG | part of a light responsive element |
| LTR | 1 | CCGAAA | cis-acting element involved in low-temperature responsiveness |
| MBS | 1 | CGGTCA | MYB Binding Site |
| MBS | 1 | CAACTG | MYB binding site involved in drought-inducibility |
| MSA-like | 1 | (T/C)C(T/C)AACGG(T/C)(T/C)A | cis-acting element involved in cell cycle regulation |
| Skn-1_motif | 2 | GTCAT | cis-acting regulatory element required for endosperm expression |
| Sp1 | 1 | CC(G/A)CCC | light responsive element |
| TATA-box | 73 | TAATA | core promoter element around -30 of transcription start |
| TATCCAT/C-motif | 1 | TATCCAT | - |
| TC-rich repeats | 1 | ATTTTCTTCA | cis-acting element involved in defense and stress responsiveness |
| TCA-element | 1 | CCATCTTTTT | cis-acting element involved in salicylic acid responsiveness |
| box S | 1 | AGCCACC | - |
| circadian | 3 | CAANNNNATC | cis-acting regulatory element involved in circadian control |

### Table S3. Accession number used in this study

| Gene name | Accession number |
| --- | --- |
| Atβ-Gal1 | NM_112225 |
| Atβ-Gal2 | NM_001339590 |
| Atβ-Gal3 | NM_119799 |
| Atβ-Gal4 | NM_125070 |
| Atβ-Gal5 | NM_103587 |
| Atβ-Gal6, | NM_125775 |
| Atβ-Gal7 | NM_122078 |
| Atβ-Gal8 | NM_179790 |
| Atβ-Gal9 | NM_128841 |
| Atβ-Gal10 | NM_125776 |
| Atβ-Gal11 | NM_119667 |
| Atβ-Gal12 | NM_118747 |
| Atβ-Gal13 | NM_127225 |
| Atβ-Gal14 | NM_120020 |
| Atβ-Gal15 | NM_001332978 |
| Atβ-Gal16 | NM_001334779 |
| Atβ-Gal17 | NM_105957 |
| Faβ-Gal1 | AJ278703 |
| Faβ-Gal2 | AJ278704 |
| Faβ-Gal3 | AJ278705 |
| Faβ-Gal4 | KR189030 |
| Mdβ-Gal1 | L29451 |
| Mdβ-Gal2 | XM_008389715 |
| Mdβ-Gal3 | XM_008368138 |
| Mdβ-Gal4 | XM_008353123 |
| Mdβ-Gal5 | XM_008340441 |
| Mdβ-Gal6 | XM_008339663 |
| Mdβ-Gal7 | XM_008395970 |
| Mdβ-Gal8 | XM_008395969 |
| Mdβ-Gal9 | XM_008393104 |
| Mdβ-Gal10 | XM_008385587 |
| Mdβ-Gal11 | XM_008379521 |
| Mdβ-Gal12 | XM_008378967 |
| Mdβ-Gal13 | XM_008360184 |
| Pbβ-Gal1 | KR024004 |
| Pbβ-Gal2 | KC855743 |
| Pbβ-Gal3 | KC855744 |
| Pbβ-Gal4 | KC855745 |
| Pbβ-Gal5 | KC855746 |
| Pbβ-Gal6 | KC855747 |
| Pbβ-Gal7 | KC855748 |
| Pcβ-Gal1 | AB086423 |
| Pcβ-Gal2 | AB086424 |
| Pcβ-Gal3 | AB086425 |
| Pcβ-Gal4 | AJ811694 |
| Ppβ-Gal1 | EF568777 |
| Ppβ-Gal2 | EF568776 |
| Ppβ-Gal3 | AY874412 |
| Ppyβ-Gal1 | AB046543 |
| Ppyβ-Gal2 | AB190363 |
| Ppyβ-Gal3 | AB190364 |
| Ppyβ-Gal4 | AB190365 |
| Ppyβ-Gal5 | AB190366 |
| Ppyβ-Gal6 | AB190367 |
| Ppyβ-Gal7 | AB190368 |
| Ppyβ-Gal8 | AB190369 |
| Slβ-Gal1 | NM_001247536 |
| Slβ-Gal2 | NM_001247369 |
| Slβ-Gal3 | NM_001247374 |
| Slβ-Gal4 | NM_001247378 |
| Slβ-Gal5 | HM627534 |
| Slβ-Gal6 | HM627535 |
| Slβ-Gal7 | HM627536 |
